# Sustainable bio-derived functional materials enabling efficient aqueous zinc-ion batteries: a mini review

**DOI:** 10.1039/d6ra07002g

**Published:** 2026-09-21

**Authors:** Lashari Najeeb ur Rehman, Glaydson Simoes dos Reis

**Affiliations:** a Laboratory of Industrial Chemistry and Reaction Engineering, Faculty of Science and Engineering, Åbo Akademi University 20500 Åbo/Turku Finland Najeeb.Lashari@abo.fi Glaydson.simoesdosreis@abo.fi; b Universidad de La Costa, CUC Calle 58 # 55–66 Barranquilla Atlántico Colombia

## Abstract

The increasing need for safe, low-cost and sustainable electrochemical power storage has spurred the development of aqueous zinc-ion batteries (ZIBs) as a possible alternative to lithium-ion batteries. Among the variety of cathode materials explored, biomass-derived carbon compounds have attracted considerable attention due to their renewable origin, tunable porosity, abundant surface functional groups, and eco-friendly synthesis methods. However, the biomass source of these materials dictates their electrochemical performance, but more significantly their chemical structure, including pore architecture, defect density, graphitization degree, heteroatom functionalization, and interfacial chemistry. This mini review provides a critical summary of the correlation between the structural properties of biomass-derived carbon materials and their Zn^2+^ storage mechanisms and emphasizes the effects of structural engineering on ion adsorption, charge transfer, diffusion kinetics, pseudocapacitive behavior, and cycling stability. Recent progress in heteroatom doping, hierarchical porous designs and metal–organic framework (MOF)-derived carbon composites are presented with focus on their roles in boosting electrochemical performance. The review does not summarize individual findings but develops structure–property–performance correlations that give essential insights for rational design of high-performance biomass-based cathodes. Finally, the current challenges, such as scalable synthesis, mechanistic understanding with *operando* characterization, structural stability, and practical device integration, are critically discussed along with future perspectives for the development of sustainable and high-energy-density zinc-ion energy storage systems.

## Abstract 2

The increasing need for safe, low-cost and sustainable electrochemical power storage has spurred the development of aqueous zinc-ion batteries (ZIBs) as a possible alternative to lithium-ion batteries. Image prepared using Microsoft Copilot.

## Graphical abstract

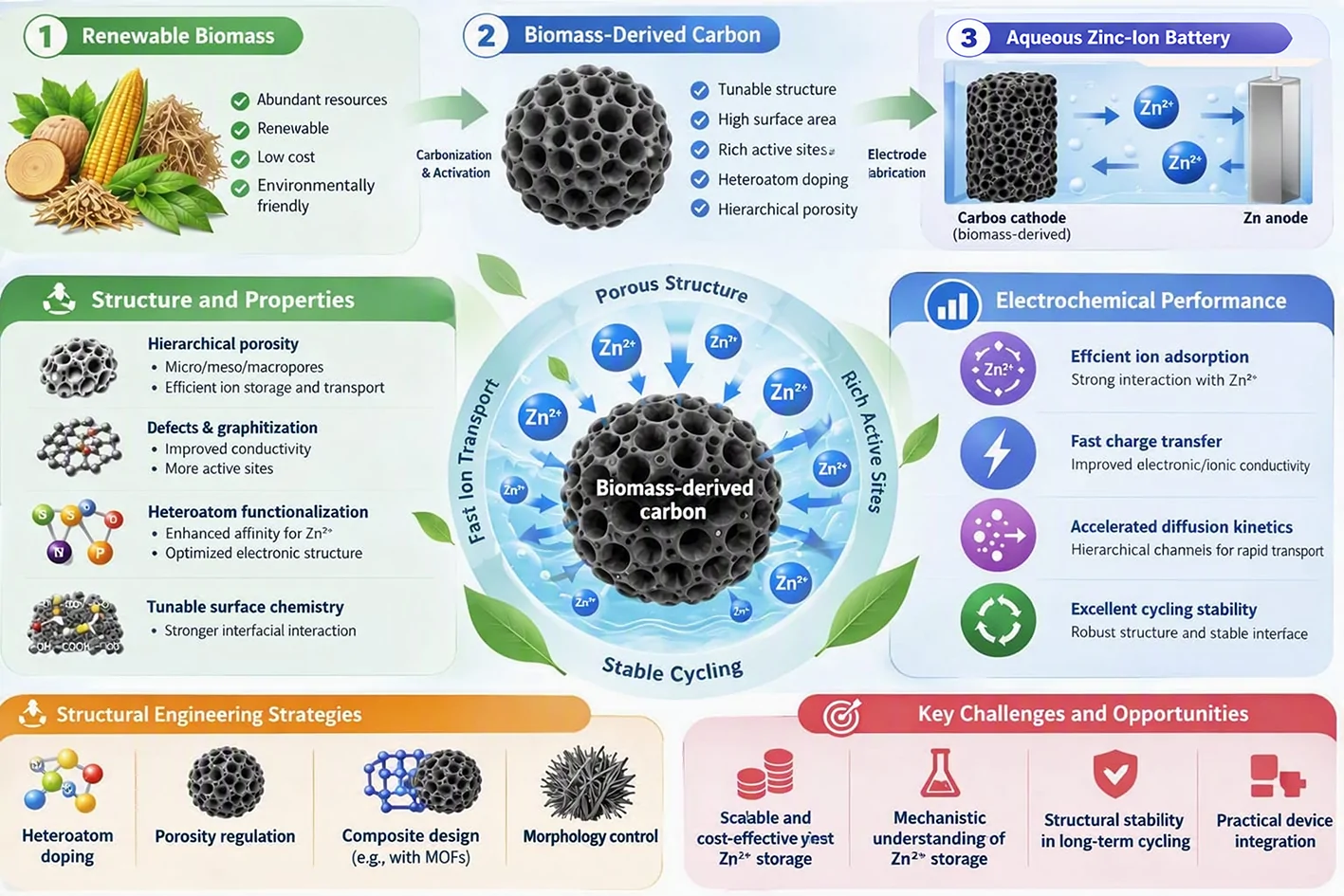

## 1 Introduction

The increasing integration of renewable energy and the demand for safer, more sustainable, and lower-cost energy storage systems have spurred widespread research into alternatives to lithium-ion batteries. There are several battery chemistries being investigated, including sodium-ion batteries, the most advanced behind LIBs. Among the emerging battery technologies, aqueous zinc-ion batteries (ZIBs) have garnered considerable attention due to Zn's natural abundance, high theoretical gravimetric capacity (820 mAh g^−1^), relatively low standard electrode potential (−0.76 V *vs.* SHE for the Zn^2+^/Zn couple), low toxicity, and the intrinsic safety of aqueous electrolytes, making Zn a promising contender for next-generation sustainable energy-storage systems.^1–3^ However, despite many advantages, practical ZIB fabrication and widespread utilization is still hampered by some important bottlenecks such as cathode structural instability, sluggish Zn^2+^ diffusion kinetics, and unwanted side-reactions at the electrode–electrolyte interface.^4,5^ To address these limitations, the rational design of advanced cathode materials that efficiently accommodate Zn^2+^ ions without compromising structural integrity or electrochemical performance is warranted.

Recent investigations have further established that the electrochemical performance of aqueous Zn-ion batteries is substantially regulated by electrode structure, interfacial chemistry, and Zn^2+^ transport kinetics. Rational management of electrode materials and their interfaces can promote Zn^2+^ storage and charge-transfer activities while minimizing structural deterioration during repeated cycles. These advances further emphasize the importance of developing structurally engineered and chemically functionalized electrode materials for achieving high-performance and durable aqueous ZIBs.^6,7^ The conventional ZIBs host materials are reported to be manganese-based oxides, vanadium-based compounds, Prussian blue analogs, and organic quinones, which are capable of reversibly intercalating Zn^2+^ ions.^8–12^ However, many of these materials suffer from intrinsic drawbacks, such as structural degradation during repeated Zn^2+^ insertion/extraction, sluggish ion diffusion kinetics, dissolvation of active species, and, in some cases, production costs and limited sustainability. In this sense, biomass-derived carbons have emerged as potentially very promising host materials for high-energy density and effective ZIBs due to their tunable porosity, easy structure modification/functionalization, suitable electrical conductivity, and very active surface chemistry that enable and boost charge transport and facilitate fast Zn^2+^ ion diffusion.^13,14^ Recent advances have shown effective strategies in developing porous carbons with different characteristics and modification (*e.g.*, heteroatom-doping, and compositing with metals and other organic molecules) to significantly improve the electrochemical activity and durability of carbon hosts over the cycling process.^15–17^ In addition to providing a conductive backbone, biomass-based carbon materials can be engineered with appropriate defects and functional groups that serve as active sites for Zn^2+^ storage.

Beyond the intrinsic structural characteristics of the biomass-based carbon, further improvement in Zn^2+^ storage can be achieved through their functionalization and hybridization. Incorporating heteroatoms (*e.g.*, nitrogen, sulfur, boron, oxygen) or coupling carbon matrices with redox-active compounds (*e.g.*, metal oxides (MnO_2_, vanadium oxides) and organic molecules) into their carbon structures can significantly enhance electrochemical reactivity and stabilize electrode structures during cycling. Moreover, biomass-derived carbons usually exhibit a high degree of structural defects and, with the right methodology, can have their interlayer spacing expanded, thereby facilitating Zn^2+^ intercalation/deintercalation processes and enhancing the capacity of ZIBs. These innovations demonstrate the versatility of biomass-derived carbon hosts for ZIB electrodes.

Overall, biomass-derived host materials offer a viable pathway to develop next-generation, sustainable, and durable batteries beyond LIBs. By integrating a biomass-compatible chemical composition with environmentally benign synthesis methods, the research has achieved notable improvements in capacity, cycling stability, and rate performance. However, to further advance ZIBs, a deeper understanding of (i) Zn^2+^ storage mechanisms, (ii) interactions between electrode/electrolyte, (iii) degradation process, (iv) optimization of scalable, sustainable fabrication processes is needed to be addressed.^18–20^ Therefore, biomass-derived carbon hosts are poised to play a critical role in enabling next-generation energy storage systems such as ZIBs that align with both sustainability and performance goals.

Biomass-derived carbon materials are promising cathode options owing to their renewable origin, environmental sustainability, widespread availability, low cost, and scalability, serving as alternatives to synthetic carbon- and metal-based materials. Porous carbon compounds with adjustable physicochemical characteristics may be synthesized from different biomass precursors, such as coconut shells, lignin, cellulose, chitosan, and agricultural leftovers. Biomass-derived carbon cathodes have exhibited high specific capacities, excellent cycling stability, and superior rate capability, mainly owing to their interconnected porous structures, improved electrical conductivity, and abundant electrochemically active sites for Zn^2+^ storage.

However, the structural properties of the biomass-derived carbons continue to significantly influence their electrochemical performance. Most biomass-derived carbons have randomly dispersed pores, nonuniform pore-size distributions, and excessive microporosity, which are typically insufficient to adequately accept hydrated Zn^2+^ ions. Moreover, the uncontrolled defect architectures and restricted interlayer spacing may hinder Zn^2+^ diffusion, thereby slow charge-transfer kinetics and limiting the exploitation of electrochemically active sites. Such restrictions suggest that merely converting biomass into porous carbon is insufficient to achieve high-performance ZIB cathodes. However, rational structural engineering, such as pore architecture optimization, defect engineering, heteroatom doping, and hybrid composite design, is necessary to tailor Zn^2+^ storage behavior and achieve maximum electrochemical performance. Li *et al.* investigated meso–microporous carbon materials with different pore structures to elucidate their influence on Zn^2+^ storage. The optimized carbon exhibited a high specific surface area of 3823 m^2^ g^−1^ and a large pore volume of 3.01 cm^3^ g^−1^, providing abundant accessible sites for Zn^2+^ storage. Importantly, appropriate pore dimensions facilitate the adsorption and accommodation of Zn^2+^ species and promote efficient ion transport within the porous carbon framework. In addition, oxygen-containing surface functional groups contribute to reversible Zn^2+^ adsorption and provide additional electrochemically active sites. The interconnected meso–microporous architecture, together with favorable electrolyte wettability, further facilitates ion and electron transport. As a result, the optimized carbon cathode exhibited a capacity of 257 mAh g^−1^ and an energy density of 199.8 Wh kg^−1^ at a power density of 77.6 W kg^−1^, while retaining 95.4% of its capacity after 10 000 cycles at 2 A g^−1^.^21^

**Fig. 1:**
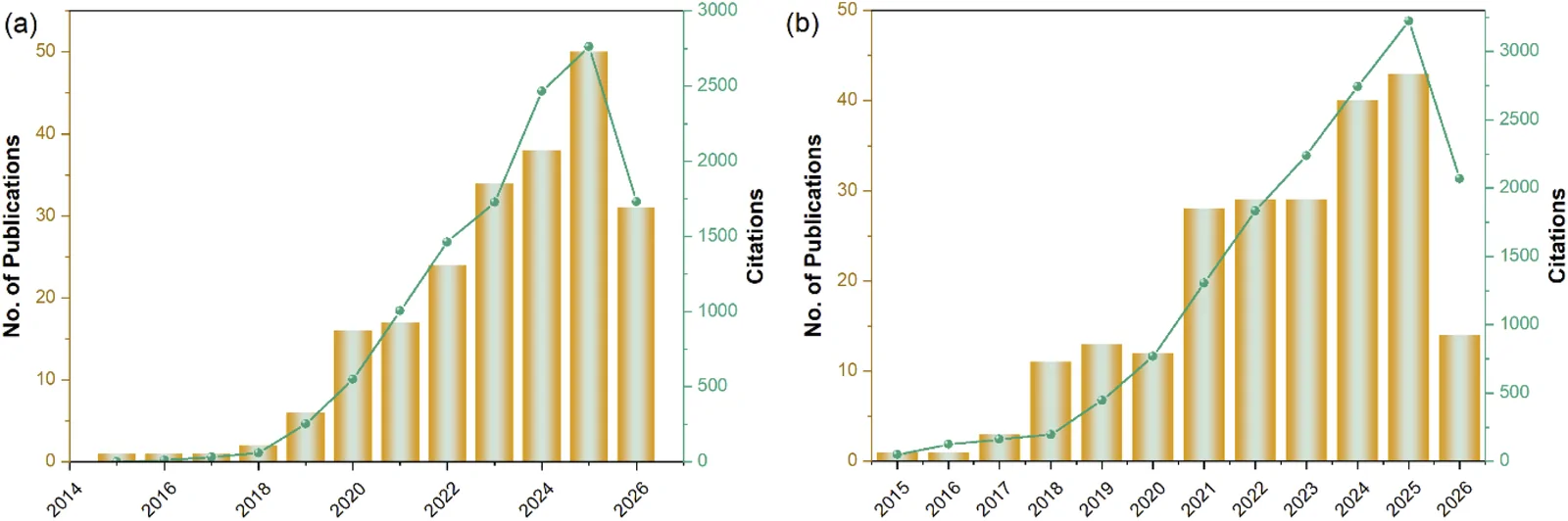
Shows the publication over span of 10 years and citation with keywords (a) biomass derived carbon for Zinc ion batteries and (b) MOF derived carbon for Zinc ion batteries. (Source: web of science).

As shown in Fig. 1 that over the span of 10 years number of publication have grown steadily but quite a few review publications have described biomass-derived carbons, heteroatom-doped carbon materials, and cathode engineering methodologies for aqueous zinc-ion batteries, most of them classify materials by biomass precursor or modification strategy. The links among chemical structure, Zn^2+^ storage methods, and electrochemical performance have received comparatively little systematic attention. Such insight is critical for identifying the fundamental structural characteristics that govern ion adsorption, diffusion kinetics, charge-transfer behavior, and long-term cycling stability, thereby enabling the rational design of next-generation biomass-derived electrodes. In this mini review, we will critically examine recent advances in biomass-derived carbon materials and MOF-derived carbon composites for aqueous zinc-ion batteries, based on structure–property–performance correlations. The impact of pore architecture, graphitization, defect engineering, heteroatom functionalization, and hybrid composite design on Zn^2+^ storage mechanisms and electrochemical behavior is highlighted. Finally, remaining scientific and technological hurdles and future research prospects towards scalable and sustainable zinc-ion energy storage devices are critically examined.

## 2 Structural features of biomass-derived carbon and how they affect the ZIBs performance

The biomass-based carbons have intrinsic porosity with the presence of micropores (<2 nm), mesopores (2–50 nm), and macropores (>50 nm), and each one of these pores play a role in the materials performance. For instance, micropores are responsible for the enhanced (high) SSA and abundant adsorption sites. Mesopores better enable the Zn^2+^ ions to be move and diffuse into the electrode, while macropores serve as reservoirs for the electrolyte/Zn^2+^ and shorten diffusion pathway.^22,23^

To achieve a better ZIBs performance, an interconnected hierarchical pore network can be more effective than having a extremely high SSA (elevated microporosity) because it can difficult the ion transportation and possibly increase irreversible side-reactions and thus reducing battery performance.

Generally, biomass-based carbons are rich in surface functionalities, which improve the electrode wettability. Biomass is rich in oxygen content, which ended up in producing carbon materials with abundant oxygen-containing groups, which may improve the interaction between aqueous electrolyte and the carbons.^24^ Functional groups are desired because they provide more active adsorption sites and make the electrode surface more hydrophilic surface.^25^ These are desired because it easy the electrolyte penetration and Zn^2+^ access. However, excessive oxygen-surface functionalities can provoke undesired side reactions.^25^

Biomass-derived carbons can have suitable interlayer spacing for ZIBs. Depending of the preparation method, the carbon materials may contain partially graphitized or turbostratic layers. A larger interlayer spacing enable a more effective Zn^2+^ intercalation. Moreover, it leads to an energetic barrier reduction, this is extremely relevant when the carbon participates directly in Zn^2+^ storage^26^ in principle, larger interlayer spacing can improve the ZIBs cycling stability in a long term.

The degree of graphitization is another feature of biomass-derived carbons that affect their electrochemical performance when applied as electrode in ZIBs.^27^ The carbon material contains sp^2^ structure, graphitic domains, and defect density in its carbon network that determine/impact its conductivity. A higher graphitization degree improves the electron transport, and provides lower internal resistance, which enhances the rate capability of the ZIBs.^28^ However, it is important to control the graphitization of carbon because graphitized carbon contains few defects and fewer chemically active sites. Therefore, instead of a highly graphitized carbon a moderate degree of disordered carbon may offer a better tradeoff between electrical conductivity and electrochemical activity. Table 1 provides a clearer and straightforward visualization of which one and how the carbon feature impact on the ZIBs performance.

**Table 1:** Structural features of the biomass-based carbons on ZIBs performance

Structural feature	Main effect	ZIB consequence
Hierarchical pores/low microporosity	Faster ion transport	Better rate capability
High SSA	More active sites	Higher specific capacity, but possibly more side reactions
Mesopores/macropores	Electrolyte penetration	Lower diffusion resistance
Graphitic domains	Electron transport	Better conductivity/rate performance
Defects/edge sites	Zn^2+^ adsorption	Increased capacity
N/O/S/P doping	Electronic/surface modification/hydrophilicity	Enhanced kinetics and wettability
Hydrophilic groups	Electrolyte affinity	Better ion accessibility
Expanded interlayer spacing	Better Zn^2+^intercalation	Enhanced Zn^2+^ storage
Robust 3D framework	Better structural integrity	Better cycling stability

## 3 Heteroatom doping as an effective strategy for boosting carbon-based cathode materials for ZIBs

**Fig. 2:**
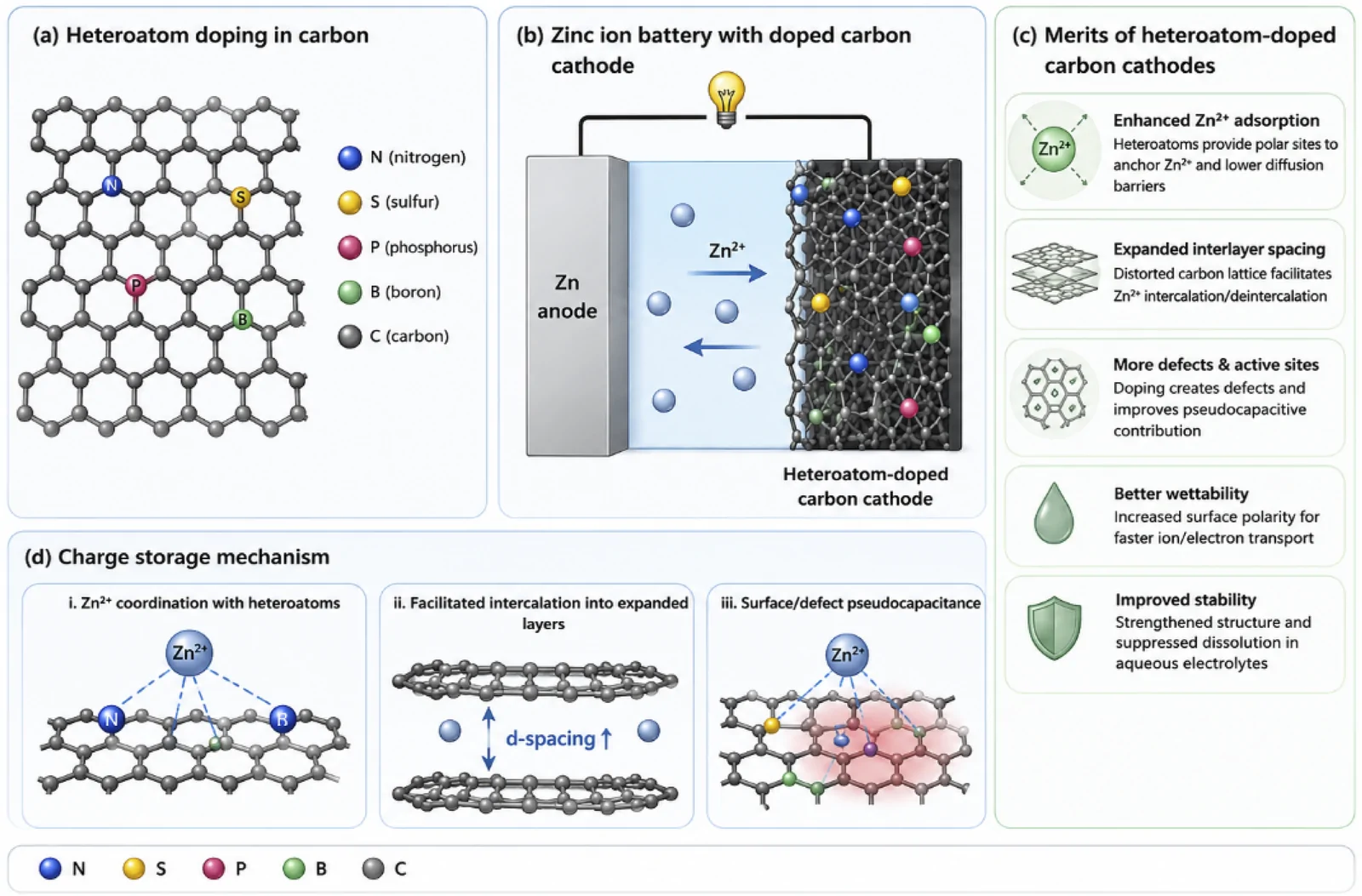
(a) Shows a carbon doped structure doped with different heteroatoms, (b) shows the schematic of a zinc ion battery with a zinc anode and a heteroatom-doped carbon as a cathode, (c) lists the merits of a heteroatom-doped carbon cathode, and (d) shows the charge storage mechanism.

Biomasses are also suitable candidates for ZIB cathodes due to their inherent chemical composition, which is rich in oxygen, nitrogen, and sulfur as shown in Fig. 2(a). After thermochemical conversion, the nitrogen-, oxygen-, and sulfur-containing functionalities inherited from biomass can modify the electronic structure, surface polarity, wettability, and defect density of carbon. These properties can generate active sites for Zn^2+^ adsorption and coordination and facilitate electrolyte penetration, Zn^2+^ desolvation, interfacial charge transfer, and ion-transport kinetics in aqueous zinc-ion systems.^29–34^ Heteroatom doping is a highly effective strategy to modify these carbons by introducing new redox sites.^15,35^ Heteroatom doping may alter the frontier molecular orbitals and the distribution of carbon charges, thereby enhancing the oxygen absorption and desorption capabilities of electrode materials. Typically, doping induces changes in the materials' electronegativity, leading to an asymmetric distribution of charge and electron spins (Fig. 2(d)). This imparts metal-like characteristics, including the formation of active sites and an altered carbon electronegativity. The redistribution of charges strengthens the interaction between oxygen molecules and carbon, thereby facilitating the redox reaction process.

### 3.1 Nitrogen doping

Nitrogen doping is a widely used technique in materials science, particularly for modifying the properties of carbon-based materials (such as graphene, carbon nanotubes, and biomass-derived carbons). Nitrogen and carbon have similar atomic radii, but they have different electron distributions and electronegativities; thus, carbon-based materials doped with N exhibit significant changes in their electronic structure while maximizing lattice matching, resulting in unique electronic properties.^36^ The nitrogen doping can provide advantages such as (i) nitrogen has one more valence electron than carbon, which introduces additional free electrons (n-type doping) on the electrode material, improving its electrical conductivity, and electrode electrochemical performances.^37^ (ii) Nitrogen doping creates active sites (*e.g.*, pyridinic, pyrrolic, and graphitic N) that enhance catalytic performance and can provide redox reaction sites to produce pseudocapacitance effect, resulting in higher capacitive materials^38^ and (iii) N-functional groups enhance the hydrophilicity, increasing the contact area between electrode and electrolyte and aiding in better electrolyte/material interaction, and improving the electrochemical performance of the energy storage devices. For instance, pyridinic nitrogen donates a p-electron to the hexagonal conjugated system, resulting in electron–donor properties in carbon-doped materials.^39^ (iv) N-doping creates incorporation, which induces structural defects, improving mechanical and chemical stability, also leading to better electrochemical properties.^40,41^

According to Zhang *et al.*, nitrogen doping can drastically increase zinc ion storage capability because the new nitrogen added sites can effectively lower the energy barrier of chemical interaction between C–O and Zn^2+^ and further boost the chemical adsorption of Zn^2+^ on the carbon electrode surface, which led to a ZIBs with outstanding energy and power densities, 107.3 Wh kg^−1^ and 24.9 kW kg^−1^, respectively. Moreover, the nitrogen-doped carbon cathode gave a remarkable capacity of 177.8 mAh g^−1^ and an ultralong-term cycling durability (99.7% capacity retention after 20 000 cycles).^42^

Li *et al.*, used a nitrogen doping process to improve the capacity and electrochemical stability of the carbon cathodes.^43^ In chemical effect, the nitrogen-doped carbon active sites can reconstruct the typical solvation structure of,^29^ and further facilitate the desolvation process. It was reported that the pyrrole and pyridine nitrogen states boost the bond length of Zn^2+^ and surrounding solvated water molecules, which, in the end, helped to suppress the zinc dendrite growth, and thus boost the electrochemical performance/stability of the ZIBs. Wei performed experiments on the preparation of nitrogen-doped porous carbon cathode for ZIBs, and the results showed a high discharge capacity of 204.7 mAh g^−1^ and an energy density of 143 Wh kg^−1^, with very high cycling stability, with 95.5% of initial capacity after 20 000 cycles.^30^

Nitrogen doping has been shown to be an effective strategy for enhancing the electrochemical performance of many energy storage systems, including ZIBs. It is reported that N-doping can enhance Zn^2+^ storage because it diminishes the energy potential barrier for C–O–Zn bond, thereby improving the chemisorption of zinc ions.^42^ N-doping introduces new N-functional groups with properties like those of oxygen groups and enables the formation of N–Zn–O bonds. These bonds are formed during the charging process, and they disappear during the discharge process.^15^

The literature contains numerous and exciting reports on the production of bio-based ZIBs using different heteroatom-doping strategies, as shown in Table 2. From these studies, different takeaways and outcomes have been achieved regarding capacity, energy density, cycling performance, and capacity retention, which highlights that (i) high heteroatom content positively influences the electrical conductivity and (ii) high electrical conductivity leads to better electrochemical performance.

**Table 2:** Contrast of the zinc ion storage properties of the different heteroatom-doped carbon-based ZIBs

Carbon source	Heteroatom type/source	Capacitance/energy density	Cycles/capacity retention	Ref.
Chitosan	Nitrogen/Fe(NO_3_)_2_	136.8 mAh g^−1^ (0.1 A g^−1^)/191 Wh kg^−1^	5 000/90.9%	31
Mushroom substrate	Nitrogen/self-doping	122.8 mAh g^−1^/122.8 Wh kg^−1^	15.000/100%	44
Commercial camphor pallets	Nitrogen/urea	268 mAh g^−1^ (1.0 A g^−1^)/106.31 Wh kg^−1^	10 000/97.08	45
Old rice	Nitrogen/urea	164.0 mAh g^−1^ (0.2 A g^−1^)/131.2 Wh kg^−1^	10 000/94.3%	46
Commercial AC	Nitrogen/melamine	105 mAh g^−1^ (0.2 A g^−1^)	25 000/89.4%	47
Bamboo	Nitrogen/NH_4_H_2_PO_4_	132.2 mAh g^−1^ (5.0 A g^−1^)/131.2 Wh kg^−1^	1 500/76.8%	48
Loofah sponges	Nitrogen/self-doping	341 mAh g^−1^ (0.2 A g^−1^)/540 Wh kg^−1^	3 500/87%	49
Sugarcane stems	Nitrogen/self-doping	198.4 mAh g^−1^ (0.2 A g^−1^)/156 Wh kg^−1^	10 000/100%	50
Orange peel	Oxygen/KOH	125.7 mAh g^−1^(1.0 A g^−1^)/69.4 Wh kg^−1^	3 000/92%	34
Anhydrous glucose	Oxygen/KHCO_3_	147 mAh g^−1^ (0.2 A g^−1^)/40.0 Wh kg^−1^	20 000/88%	51
Commercial AC	Oxygen/HNO_3_	132.7 mAh g^−1^ (1.0 A g^−1^)/82.0 Wh kg^−1^	10 000/87.6%	52

### 3.2 Oxygen doping

Oxygen doping has attracted significant attention due to its abundance, non-toxicity, and universality.^53^ Additionally, oxygen can be readily used to modify carbon materials, yielding various oxygen-containing functional groups within their structures. These oxygen functionalities provide excellent reactive sites for energy storage, enabling redox reactions with electrolyte ions. These reactions can be highly efficient and reversible, adding an important pseudo-capacitive effect and thus enhancing the electrochemical performance of the oxygen-doped carbon materials.^54^ Other important effects of oxygen-doping are that oxygen functionalities enhance the surface hydrophilicity (and wettability), which facilitates more active sites for charge storage,^55^ as well as significantly improve the electrical conductivity of carbon materials through Faraday reactions.^56^ Recent works on doping/incorporating O elements in carbon structures have shown that Zn^2+^ ion storage is largely improved not only due to the higher electrode's hydrophilicity/wettability, but also because the O-groups act with Zn^2+^ ions to form C–Zn–O bonds and thereby improve the pseudo-capacitance effect of the ZIBs cells.^57,58^

Yin *et al.*, employed oxygen-rich porous carbon as a cathode in an aqueous zinc-ion system.^58^ The enhanced electrochemical performance was attributed to the combination of a hierarchically porous carbon framework and oxygen-containing surface sites. The porous structure improved electrolyte accessibility and shortened ion-transport pathways, while the oxygen functionalities provided sites for reversible Zn^2+^ adsorption or coordination and increased the surface-controlled contribution to charge storage. The results showed outstanding capacity and energy density due to the EDLC contribution from the porous carbon structure and an additional pseudo-capacitive contribution from the different oxidation states of oxygen functional groups. The ZIBs delivered a capacity of 179.8 mAh g^−1^ in a wide voltage window of 0–1.9 V, and an energy density of 104.8 Wh kg^−1^ at 48.8 kW kg^−1^. The ZIBs exhibited a very long lifespan (30 000 cycles and 99.2% capacity retention).

Oxygen-containing functional groups can influence aqueous Zn^2+^ storage by modifying the polarity, wettability, and coordination chemistry of the carbon surface. Carbonyl, hydroxyl, ether, epoxy, and carboxyl-related groups may provide adsorption or coordination sites for hydrated or partially desolvated Zn^2+^. These interactions can facilitate Zn^2+^ accumulation at the electrode–electrolyte interface and contribute to rapid surface-controlled charge storage. In some oxygen-functionalized carbons, reversible C–O–Zn interactions have been proposed to account for the Zn^2+^-associated faradaic contribution. However, the activity of each oxygen group depends on its chemical environment, accessibility, and stability within the operating voltage window of the aqueous electrolyte. The influence of oxygen functionalization on aqueous Zn^2+^ storage depends on the type and accessibility of the surface groups. Carbonyl- and carboxyl-related groups can provide polar coordination environments for Zn^2+^, whereas hydroxyl, ether, and epoxy groups can improve surface wettability and electrolyte accessibility.^56–58^ These oxygen-containing sites may facilitate reversible Zn^2+^ adsorption, partial desolvation, and interfacial charge transfer. Therefore, their contribution should be described as surface-controlled Zn^2+^ storage or Zn^2+^-coupled charge transfer rather than as general pseudocapacitance.^57,58^ The literature shows that the carbon surface oxygen-functional groups can be classified into acidic groups (which include carboxyl and carboxylic anhydride), basic groups (which include quinone and carbonyl groups, and neutral groups (also called weakly acidic groups, which include hydroxyl, epoxy, and ether groups. The acidic functionalities can play an important role in alkaline electrolytes.^39^ These oxygen-functional groups in carbon materials act as electron donor–acceptor sites, serving as active sites with pseudo-capacitive characteristics and thereby boosting the energy density of the ZIBs to a certain extent.

### 3.3 Sulfur doping

Sulfur is one of the most common dopants for carbon materials; it has an electronegativity higher than that of carbon and can act as an electron donor.^59–62^ The incorporation of sulfur atoms into the carbon structure enhances the electron density and surface polarization of the doped material, which promotes a better charge transfer, and enhances their electrochemical properties.^59^ Sulfur doping modifies the local electronic structure of carbon and introduces defects and polarized sulfur-containing sites that can enhance Zn^2+^ adsorption and interfacial charge transfer.^59^ Because sulfur has a larger atomic radius than carbon, its incorporation may also expand or distort the carbon lattice, thereby improving electrolyte accessibility and reducing Zn^2+^ diffusion limitations. The main potential contributions of sulfur doping to aqueous Zn^2+^ storage are: (i) expansion or distortion of the carbon framework, which may facilitate Zn^2+^ transport; (ii) creation of defects and polarized C–S-containing sites for Zn^2+^ adsorption; (iii) improvement of electrode wettability and electrolyte penetration;^33^ and (iv) possible reversible Zn^2+^-associated reactions at sulfur-containing sites. The latter mechanism should only be assigned when supported by electrochemical, computational, or spectroscopic evidence.

### 3.4 Phosphorous doping

As with other non-metal dopants, phosphorus doping is also seen as a highly effective strategy for modifying and tailoring the physicochemical/electrochemical features of carbon materials for ZIBs. Phosphorus has a larger atomic radius than carbon and can introduce structural defects by distorting the carbon lattice and modifying the electronic configuration of the carbon framework. Phosphorus-based surface functionalities on carbon surfaces can increase surface polarity and improve electrolyte/surface contact and wettability, facilitating electrolyte penetration/diffusion and boosting Zn^2+^ transport. Moreover, P-doping creates new active sites for Zn^2+^ adsorption that, when combined with a hierarchical porous structure, promote faster ion diffusion and electron transfer, thereby improving the electrode's rate capability and stability.

Recent studies further demonstrate that the beneficial role of phosphorus-containing carbon materials in aqueous ZIBs is not limited to cathode modification but can also be extended to interfacial and separator engineering. Fan Huailin, *et al.* developed P-doped honeycomb-like porous carbon as a cathode for Zn-ion storage and demonstrated that P doping enhanced the pseudocapacitive contribution by promoting the chemical adsorption of Zn ions and improving the stability of Zn-ion storage.^62^ More recently, Huang *et al.* employed a P-doped carbon protective layer on the Zn anode, where the modified interface enhanced conductivity and Zn^2+^ diffusion kinetics, lowered the nucleation overpotential, and suppressed undesirable side reactions.^61^ Similarly, Sun Zhichao *et al.* prepared low-cost biomass-derived carbon through carbonization and phosphitylation and used the P-containing carbon to modify a glass-fiber separator, effectively regulating Zn deposition and improving the cycling stability of aqueous ZIBs.^13^ Furthermore, Zou Yuefei, *et al.* developed a phosphorus-doped carbon nitride/cellulose nanofibril (PCN@CNF) interlayer containing abundant N- and P-functionalities. The resulting zincophilic interface regulated Zn^2+^ flux and homogenized the electric and ionic fields, thereby suppressing Zn dendrite formation and interfacial side reactions.^60^ Collectively, these studies demonstrate that rational phosphorus functionalization can contribute to ZIB performance through multiple mechanisms, including enhanced Zn-ion adsorption, improved interfacial ion transport, regulated Zn deposition, and suppression of parasitic reactions. Therefore, careful control of the phosphorus chemical environment and its location within the electrode–electrolyte system is important for exploiting the full potential of P-functionalized carbon materials in durable aqueous ZIBs.

The effect of phosphorus doping depends on its concentration and bonding configuration. At an appropriate concentration, P doping can introduce Zn^2+^-affinitive defects, modify the electronic structure, and expand or distort the carbon framework, potentially improving Zn^2+^ adsorption and transport. Excessive phosphorus incorporation may generate unstable defects or poorly conductive phosphorus-containing species, increasing charge-transfer resistance and weakening structural stability. Therefore, the P content and chemical configuration should be optimized to promote reversible Zn^2+^ storage rather than merely maximizing the total phosphorus concentration.

## 4 MOF-derived carbon composites in aqueous zinc-based energy-storage systems

### 4.1 MOF-derived materials for aqueous zinc-ion batteries

**Fig. 3:**
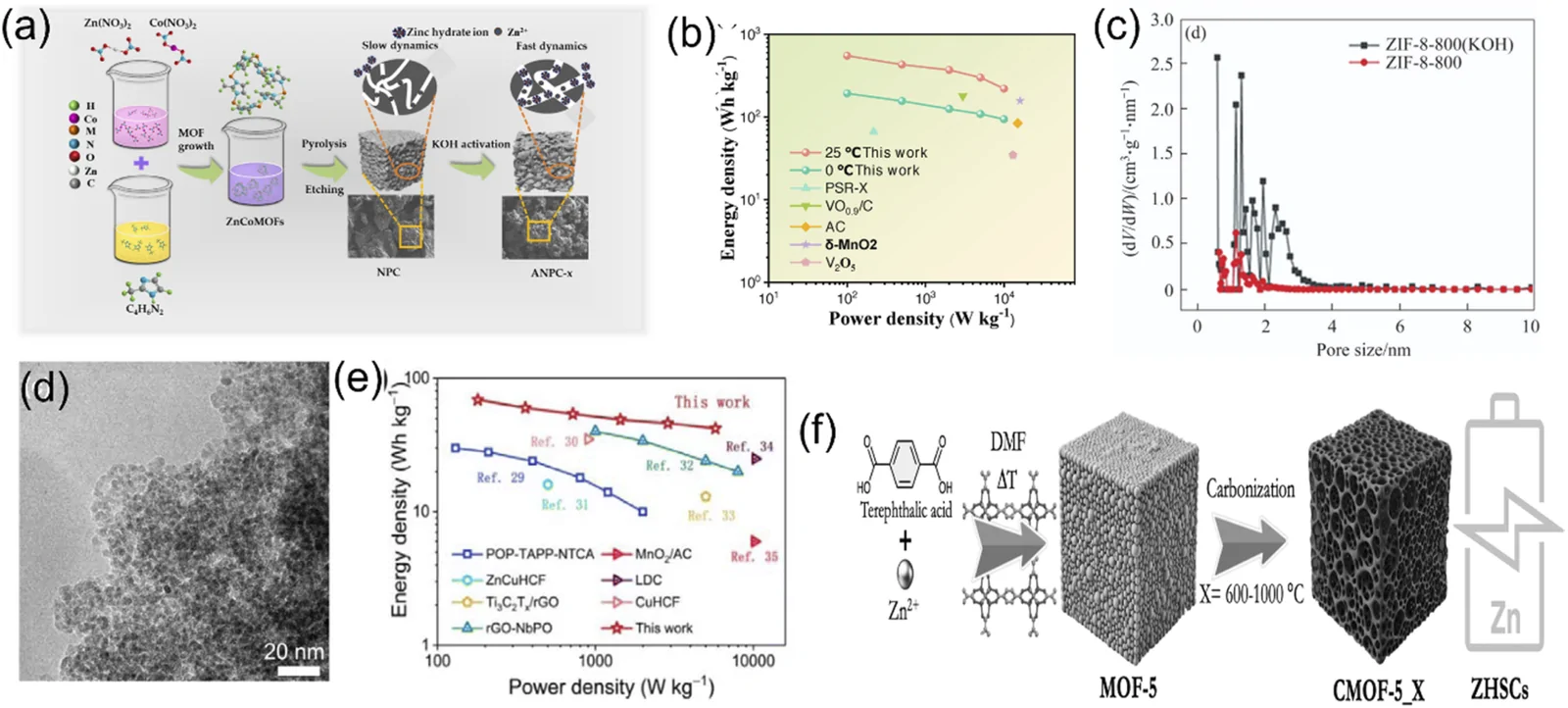
(a) Preparation route schematic of ANPC. (b) Ragone plot of several electrode materials for ZHCs (c) the pore size distribution of ZIF-8-800 and ZIF-8-800 (KOH), inset N_2_ adsorption/desorption isotherms. (d) TEM image and (e) the Ragone plot comparison between different supercapacitors and NC@ZrO_2_ composite-based ZHSs. (f) Synthetic pathway of CMOF-5.

MOF-derived carbons have been investigated in several aqueous zinc-based devices, including zinc-ion batteries (ZIBs), zinc-ion capacitors (ZICs), and zinc hybrid supercapacitors (ZHSs). These systems must be considered separately because their electrode reactions and reported performance metrics are different. In ZIBs, the cathode generally stores Zn^2+^ through insertion, adsorption, coordination, or conversion reactions, and performance is commonly reported as specific capacity in mAh g^−1^. In ZICs and ZHSs, Zn plating/stripping occurs at the negative electrode, whereas the porous positive electrode commonly stores ions through surface-controlled adsorption or fast redox reactions; performance is therefore frequently reported as capacitance in F g^−1^, energy density, and power density. Conventional symmetric supercapacitors do not contain a Zn electrode and are not considered direct evidence of Zn^2+^ storage. The systems included in this section and Table 3 are consequently classified according to their device configuration before their electrochemical performance is discussed. For instance, He *et al.*^63^ developed a MOF-derived porous carbon material (ANPC) that delivered a high specific capacity of 173.6 mAh g^−1^ at 0.5 A g^−1^ and retained 92% of its initial capacity after 40 000 cycles at 10 A g^−1^ (Fig. 3(a). Similarly, Zhang *et al.*^64^ synthesized a highly porous three-dimensional Mn_3_O_4_/C composite derived from Mn-BDC, which exhibited outstanding energy and power densities of 464.5 Wh kg^−1^ and 100 W kg^−1^, respectively (Fig. 3(b)). Liu *et al.*^65^ employed ZIF-8 to fabricate N, O co-doped porous carbon with a high specific surface area (2814.67 m^2^ g^−1^) and an *I*_G_/*I*_D_ ratio of 1.0 during carbonization, achieving a self-doped structure that delivered an energy density of 125 Wh kg^−1^ and a power density of 79 W kg^−1^.

**Table 3:** The following table summarizes representative MOF-derived carbon composites for zinc-ion energy storage^a^

	Composite system	Precursors	Performance metric	Key features	Ref.
ZIB	ANPC	2-Methylimidazole + Zn and Co precursor	71.52Wh kg^−1^ at 9 kW kg^−1^	Coral-like porous	63
ZIC or ZHS	Mn_3_O_4_/C	Terephthalic acid (H_2_BDC)+ Mn precursor	464.5 Wh kg^−1^ at 100 W kg^−1^	Large specific surface area and adjustable pore structures	64
ZIC	ZIF-8-800	ZIF-8 + KOH, Fe(NO_3_)_3_ and H_3_BO_3_ activators	125 Wh kg^−1^ and a power density of 79 W kg^−1^	Diverse pore structure and plentiful defect sites	65
Zinc-ion hybrid supercapacitor	NC@ZrO_2_	UiO-66-NH_2_ MOF + ZrO_2_	69 Wh kg^−1^ and 5760 W kg^−1^	Improved chemical adsorption and accelerated Zn^2+^-storage kinetics	66
Zinc-ion battery	V_2_O_3_/C	ZIF-8 + V precursor	270 mAh g^−1^ at 500 mA g^−1^	Enhanced electrical conductivity and structural stability	67
Zinc-ion hybrid supercapacitor	MoO_2_@Cu@C	POM@MOF	2.58 Wh kg^−1^ at 86.8 W kg^−1^	Improved dispersity and porosity of polybasic composite	68
Zinc-ion hybrid supercapacitor (ZHS)	BCNP	Co_4_/Ni_6_-BTC MOF + Na_3_PO_4_·12H_2_O	376.5 Wh kg^−1^ at 1.66 kW kg^−1^	Fast electrolyte feeding, ample volume space and rich active center	69
Zinc-ion capacitor	HCSt_6_	PDA@mSiO_2_ + TEOS	85.3 Wh kg^−1^ and 16.0 kW kg^−1^	Possess large cavity structure and an appropriate proportion of mesoporous and micropores	70

a Note: ZIB, zinc-ion battery; ZIC, zinc-ion capacitor; ZHS, zinc hybrid supercapacitor. Specific capacity, specific capacitance, energy density, and power density are reported under different testing conditions and are not directly comparable. Meaningful comparison also requires consistent consideration of the electrolyte, voltage window, current density, active-material loading, and mass basis used in the calculation. Conventional symmetric-supercapacitor results are excluded because they do not involve a Zn electrode or directly demonstrate Zn^2+^ storage.

### 4.2 MOF-derived materials for zinc-ion capacitors and zinc hybrid supercapacitors

MOF-derived porous carbons and carbon composites have also been employed in zinc-ion capacitors and zinc hybrid supercapacitors. In these devices, Zn plating/stripping generally occurs at the negative electrode, whereas the porous positive electrode stores charge through surface-controlled ion adsorption or rapid redox reactions. Consequently, capacitance, energy density, and power density reported for these devices should not be directly compared with the specific capacities of battery-type Zn-ion cathodes. MOF-derived porous carbons and carbon composites have also been employed in zinc-ion capacitors and zinc hybrid supercapacitors. In these devices, Zn plating/stripping generally occurs at the negative electrode, whereas the porous positive electrode stores charge through surface-controlled ion adsorption or rapid redox reactions. Consequently, capacitance, energy density, and power density reported for these devices should not be directly compared with the specific capacities of battery-type Zn-ion cathodes. Wang *et al.*^66^ utilized UiO-66-NH_2_ as a precursor to synthesize a nitrogen-doped porous carbon and ZrO_2_ composite (NC@ZrO_2_) as a cathode material. The resulting zinc hybrid supercapacitor (ZHS) exhibited a maximum energy density of 69 Wh kg^−1^ and a peak power density of 5760 W kg^−1^. Recently, Kukułka *et al.*^71^ investigated the carbonization of MOF-5 at different temperatures to explore the relationship between the material's physicochemical characteristics and electrochemical performance. The sample carbonized at 1000 °C exhibited the highest performance, with a specific capacitance of 268.93 F g^−1^, an energy density of 166.9 Wh kg^−1^, and a power density of 9234 W kg^−1^. These enhancements were attributed to increased porosity, improved graphitization, and reduced charge transfer resistance.

Furthermore, Park *et al.*^67^ synthesized a V_2_O_3_/carbon (V_2_O_3_/C) composite using ZIF-8 as both a sacrificial template and an *in situ* carbon source (Fig. 2(c)). The resulting material exhibited good Zn^2+^ transport kinetics, with a diffusion coefficient (D_Zn_^2+^) of 6.2 × 10^−11^ cm^2^ s^−1^. Alternatively, Mo-based polyoxometalates (POMs) were implanted into Cu-MOFs and then transformed by thermal treatment to MoO_2_@Cu@C composites, which showed remarkable cycling stability with 91% capacity retention over 5 000 cycles.^68^ The uniform dispersion of active nanoparticles within the MOF-derived carbon matrix was found to mitigate agglomeration, thereby increasing the number of accessible electroactive sites and enhancing overall electrochemical performance.

Another promising approach to the design of MOF-derived carbon materials involves developing core–shell architectures, in which the core serves as a conductive scaffold that provides mechanical stability, while the shell contributes to pseudocapacitive behavior. A hybrid supercapacitor device constructed from a BCNP//RGO-Zn configuration delivers a high energy density of 376.5 Wh kg^−1^ at a power density of 1.658 kW kg^−1^.^69^ In a parallel strategy, hollow MOF-derived carbon structures have gained attention for their high surface area, shorter ion/electron diffusion pathways, and structural adaptability to accommodate volume fluctuations during cycling. For example, Liang *et al.* fabricated carbon-doped hollow nickel–cobalt phosphate microspheres *via* a Kirkendall-effect-driven transformation of a MOF precursor. The resulting MCNPha compound exhibited an energy density of 238.18 Wh kg^−1^ at a power density of 1.70 kW kg^−1^.^72^

Furthermore, Du *et al.* employed polydopamine (PDA) nanospheres as both carbon and nitrogen sources to synthesize micro- and mesoporous double-shell hollow carbon spheres *via* spatially confined pyrolysis. The produced carbon spheres demonstrated a high specific capacitance of 389 F g^−1^ in a symmetric supercapacitor configuration and 260 F g^−1^ in a zinc-ion capacitor (ZIC), along with excellent cycling stability, retaining 99.3% of their initial capacitance after 30 000 charge/discharge cycles.^70^

Overall, MOF-derived materials perform different functions in battery-type and capacitor-type aqueous zinc systems. In ZIB cathodes, the MOF-derived composite should be evaluated according to its ability to support reversible Zn^2+^ storage, expressed primarily through specific capacity, reaction kinetics, rate capability, and capacity retention. In ZICs and ZHSs, the porous positive electrode generally provides surface-controlled charge storage, while the Zn electrode undergoes plating/stripping; these devices are evaluated primarily using capacitance, energy density, power density, and capacitance retention. Separating these device classes prevents inappropriate numerical comparisons and clarifies the relationship between the MOF-derived structure and its actual electrochemical function.

## 5 Conclusions and future perspective

The physicochemical and structural properties, including specific surface area, pore structure, graphitization degree and structural defects, surface functional groups and heteroatom doping, fundamentally determine the electrochemical performance of biomass-derived carbon materials. These coupled features facilitate charge transfer, promote ion diffusion, boost pseudocapacitive contributions and improve the long-term cycling stability during Zn^2+^ adsorption and storage behavior. Recent progress in structural engineering, particularly in heteroatom doping, hierarchical porous structures and MOF-derived carbon composites, has markedly improved the capacity, rate capability and durability of biomass-based cathodes. Also, the establishment of structure–property–performance relationships has given useful information to rationally design next generation biomass-derived carbon electrodes. However, the practical implementation of these systems is still challenged by the intrinsic variability of biomass precursors, a limited understanding of Zn^2+^ storage mechanisms, structural degradation during long-term cycling, and the difficulty of scalable and reproducible synthesis.

Further work should be directed towards the development of standardized synthesis protocols, the use of advanced *operando* characterization techniques and the incorporation of predictive design strategies to improve the understanding and optimization of electrochemical processes. Moreover, improvements in electrode engineering, electrolyte compatibility and scalable manufacturing approaches should be adopted to translate laboratory-scale materials into practical zinc-ion batteries. A better understanding of the synergistic effects of hierarchical porosity, defect engineering, and multi-heteroatom doping (*e.g.*, N, S, P, B, and O) may open new avenues for simultaneously improving ion transport, electronic conductivity, and pseudocapacitive behavior. In general, biomass-derived carbon materials are a promising class of sustainable, high-performance and cost-effective electrode materials for aqueous zinc-ion batteries, offering great potential to promote environmentally responsible and commercially viable energy storage technologies.

## Conflicts of interest

The authors declare that they have no known competing financial interests or personal relationships that could have appeared to influence the work reported in this paper.

## Data Availability

No primary research results, so ware or code have been included and no new data were generated or analyzed as part of this review
